# Mesenchymal stromal cells protect against vascular damage and depression-like behavior in mice surviving cerebral malaria

**DOI:** 10.1186/s13287-020-01874-6

**Published:** 2020-08-26

**Authors:** Maiara N. Lima, Helena A. Oliveira, Paula M. Fagundes, Vanessa Estato, Adriano Y. O. Silva, Rodrigo J. R. X. Freitas, Beatriz A. B. R. Passos, Karina S. Oliveira, Camila N. Batista, Adriana L. Vallochi, Patricia R. M. Rocco, Hugo C. Castro-Faria-Neto, Tatiana Maron-Gutierrez

**Affiliations:** 1 Laboratory of Immunopharmacology, Oswaldo Cruz Institute, Oswaldo Cruz Foundation, Fiocruz, Av. Brasil, 4365, Pavilhão 108, sala 45, Manguinhos, Rio de Janeiro, RJ 21040-360 Brazil; 2 Laboratory of Pulmonary Investigation, Carlos Chagas Filho Institute of Biophysics, Federal University of Rio de Janeiro, Rio de Janeiro, Brazil; 3 National Institute of Science and Technology for Regenerative Medicine, Rio de Janeiro, RJ Brazil; 4 National Institute of Science and Technology on Neuroimmunomodulation, Rio de Janeiro, RJ Brazil

**Keywords:** Malaria, Mesenchymal stromal cells, Blood-brain barrier, Depression

## Abstract

**Background:**

Malaria is one of the most critical global infectious diseases. Severe systemic inflammatory diseases, such as cerebral malaria, lead to the development of cognitive and behavioral alterations, such as learning disabilities and loss of memory capacity, as well as increased anxiety and depression. The consequences are profound and usually contribute to reduce the patient’s quality of life. There are no therapies to treat the neurological sequelae of cerebral malaria. Mesenchymal stromal cells (MSCs) may be an alternative, since they have been used as therapy for neurodegenerative diseases and traumatic lesions of the central nervous system. So far, no study has investigated the effects of MSC therapy on the blood-brain barrier, leukocyte rolling and adherence in the brain, and depression like-behavior in experimental cerebral malaria.

**Methods:**

Male C57BL/6 mice were infected with *Plasmodium berghei* ANKA (PbA, 1 × 10^6^ PbA-parasitized red blood cells, intraperitoneally). At day 6, PbA-infected animals received chloroquine (25 mg/kg orally for seven consecutive days) as the antimalarial treatment and were then randomized to receive MSCs (1 × 10^5^ cells in 0.05 ml of saline/mouse) or saline (0.05 ml) intravenously. Parasitemia, clinical score, and survival rate were analyzed throughout the experiments. Evans blue assay was performed at 6, 7, and 15 days post-infection (dpi). Behavioral tests were performed at 5 and 15 dpi. Intravital microscopy experiments and brain-derived neurotrophic factor (BDNF) protein expression analyses were performed at 7 dpi, whereas inflammatory mediators were measured at 15 dpi. In vitro, endothelial cells were used to evaluate the effects of conditioned media derived from MSCs (CMMSC) on cell viability by lactate dehydrogenase (LDH) release.

**Results:**

PbA-infected mice presented increased parasitemia, adherent leukocytes, blood-brain barrier permeability, and reduced BDNF protein levels, as well as depression-like behavior. MSCs mitigated behavioral alterations, restored BDNF and transforming growth factor (TGF)-β protein levels, and reduced blood-brain barrier dysfunction and leukocyte adhesion in the brain microvasculature. In a cultured endothelial cell line stimulated with heme, CMMSC reduced LDH release, suggesting a paracrine mechanism of action.

**Conclusion:**

A single dose of MSCs as adjuvant therapy protected against vascular damage and improved depression-like behavior in mice that survived experimental cerebral malaria.

## Background

Cerebral malaria is a significant complication of *Plasmodium falciparum* infection. Malaria is considered a neglected disease and a leading public health challenge [1]. Approximately 25% of survivors may develop long-term cognitive alterations [2, 3] including memory and learning impairment and behavioral changes, such as anxiety and depression [4, 5]. Inflammatory mediators and accumulation of leukocytes are known modulators of blood-brain barrier (BBB) permeability [6], which may lead to cognitive dysfunction in experimental models of infectious diseases, such as malaria [7, 8] and sepsis [9]. Even with successful antimalarial treatment, cerebral malaria survivors can present cognitive and behavioral deficits. These neurological damages can have a critical impact on educational outcomes and socioeconomic status over time, and there is no treatment or effective prophylaxis; thus, new interventions are needed.

In our previous study, we were the first to demonstrate that mesenchymal stromal cell (MSC) administration protected BBB disruption and decreased neuroinflammation after cecal ligation and puncture (CLP) induced sepsis. Additionally, MSC therapy improved spatial and aversive memory and anxiety-like behavior in surviving septic mice [9]. Based on the aforementioned, we hypothesized that MSC therapy is a promising alternative in the prevention and reversal of neurological deficits in infectious diseases. In the present study, MSCs were administered as an adjuvant therapy that could mitigate malaria-induced endothelial dysfunction and behavioral impairments that cause cognitive and behavioral disabilities in survivors. In a cerebral malaria experimental model, we evaluated depression-like behavior, BBB integrity, rolling and adhesion of leukocytes in cerebral microcirculation, and inflammatory mediators.

## Methods

### Isolation and expansion of mesenchymal stromal cells

SCs were isolated from 8-week-old C57BL/6 femur and tibia bone marrows, as previously described [10]. Cells were used in the study between the third and fifth passages. For flow cytometry characterization, MSCs were harvested with Accutase (Stemcell Technology, Vancouver, BC, Canada), stained, and immediately acquired on a FACSCalibur system (BD Biosciences Pharmingen, San Diego, CA). The following monoclonal antibodies were used: anti-mouse CD29-PE (eBioscience, San Diego, CA), CD45 PE (Biolegend, San Diego, CA, USA), CD11b FITC (Biolegend, San Diego, CA), CD14 PE (BD Biosciences Pharmingen, San Diego, CA), rat anti-mouse CD90 purified (eBioscience, San Diego, CA) and anti-rat Alexa Fluor 488 (Invitrogen, Waltham, MA), rat IgG2b k Isotype Control FITC (eBioscience, San Diego, CA), rat IgG1 k Isotype Control PE (eBioscience San Diego, CA), and rat IgG2b k Isotype Control PE (eBioscience, San Diego, CA). Data were analyzed using the FlowJo software, version 10.5.3 (Becton Dickinson and Company, Franklin Lakes, NJ).

### Experimental protocol

C57BL/6 adult male mice were inoculated intraperitoneally (i.p.) with 0.2 mL suspension of 10^6^ *Plasmodium berghei* ANKA (PbA)-parasitized red blood cells. In the control group, mice were inoculated with 10^6^ non-parasitized red blood cells (control group, C). At 6 days post-infection (dpi), PbA-infected mice received chloroquine (CQ, Sigma-Aldrich, St. Louis, MO) (25 mg/kg orally in 0.2 mL saline) for seven consecutive days and were further randomized to receive sterile saline (SAL, 0.05 mL) or MSCs (10^5^ cells/mouse in 0.05 mL saline) into the jugular vein. The progression of the experimental model was evaluated by measuring parasitemia, clinical score, body and spleen weights, and survival up to 15 dpi. Parasitemia was measured by blood smear staining analysis. The clinical score was performed based on observations of parameters in a modified SmithKline Beecham, Harwell, Imperial College, Royal London Hospital, phenotype assessment (SHIRPA) protocol [8], where clinical signs, including piloerection, curved trunk, alterations in gait, seizures, limb paralysis, coma, respiratory rate, skin color alterations, heart rate, lacrimation, palpebral closure, decreased grip strength, limb, abdominal and body tone, and body temperature alterations were observed [11]. Evaluators were blinded to group assignment. Mice were given ad libitum access to food and water and monitored twice a day for signs of suffering attributable to the experimental model. If any signs of pain were observed, the animal was euthanatized and removed from the experiment. Our institution has an environmental enrichment program for all experimental animals, including cage enrichment accessories such as a mouse swing and cottage, which helps mitigate anxiety and stress reactivity.

### Tail suspension test

Tail suspension test was performed at 5 and 15 dpi. Immobility, the dependent measure of behavioral despair, was used to evaluate depressive-like behavior. Mice were attached to a support using tape placed 1 cm from the tip of their tales, raised to a height of 15 cm, and recorded for 6 min. Immobility time was analyzed in the final 4 min only [12]. Evaluators were blinded to group assignment.

### Forced swim test

Forced swim test was performed at 5 and 15 dpi. Mice were individually placed in a cylindrical tank (30 cm in diameter ×  40 cm height) filled to a height of 20 cm with water (25 ± 2 °C), so their tails could not touch the bottom of the cylinder. Mice were recorded and each test lasted 6 min. Time of immobility (absence of movement to stay afloat with exception to leg kicks) was analyzed in the final 4 min only [13]. Evaluators were blinded to group assignment.

### Cytokine and neurotrophin protein levels in the brain

Interleukin (IL)-1β, IL-6, IL-10, and transforming growth factor (TGF)-β were measured in total brain homogenates at 15 dpi, and brain-derived neurotrophic factor (BDNF) was measured in cortex and hippocampus homogenates at 7 dpi. We used commercially available ELISA kits and followed the manufacturers’ instructions (R&D Systems, Minneapolis, MN, and Abcam, Cambridge, UK).

### Evans blue dye assay

Evans blue dye assay was performed at 6, 7, and 15 dpi. Animals were anesthetized by intraperitoneal (i.p.) injection of ketamine (100 mg/kg) and xylazine (10 mg/kg) and injected with 2% Evans blue dye into the retro-orbital venous sinus. After 1 h, mice were euthanized with ketamine (300 mg/kg) and xylazine (30 mg/kg) i.p. and their brains were removed and placed in formamide. After 24 h of incubation at 37 °C, Evans blue dye (μg/mg of brain weight) was quantified by spectrophotometry.

### Immunofluorescence microscopy

Mice were anesthetized ketamine (300 mg/kg) and xylazine (30 mg/kg) i.p., and cardiac perfusion was performed with a peristaltic pump. Their brains were dissected and post-fixed in 4% paraformaldehyde. After embedded in optimal cutting temperature (OCT) compound, frozen longitudinal sections (12-μm thick) through the subventricular zone were prepared. The nuclei were stained with mounting medium containing DAPI (4′,6-diamidino-2-phenylindole) (Vector Laboratories). The slides were photographed at × 40 magnification on the Apotome microscope (Zeiss) coupled to a digital camera (AxioCam, Zeiss).

### Intravital microscopy through cranial window

Cerebral microcirculation in mice was assessed as previously described [14]. Briefly, animals were anesthetized with ketamine (100 mg/kg) and xylazine (10 mg/kg) i.p. at 7 dpi and placed in a stereotaxic frame. A midline skin incision exposed the left parietal bone and a craniotomy was performed over the temporal parietal bone (5 mm lateral incision between the coronal and the lambdoid sutures) with a hand-held drill to expose the cortical microcirculation. The cranial window was suffused with artificial cerebrospinal fluid (in mmol: NaCl, 132; KCl, 2.95; CaCl2, 1.71; MgCl2, 0.64; NaHCO3, 24.6; dextrose, 3.71; and urea, 6.7; at 37 °C, pH 7.4). Animals were placed under an upright fixed-stage intravital microscope equipped with a LED lamp (Zeiss, model SCOPE AXIO, Oberkochen, Germany) coupled to a Zeiss AxioCam with an × 20 water immersion objective producing a final magnification of × 200. Images were captured and processed using ZEN software (Zeiss, Oberkochen, Germany).

The visualization of the brain microvascular surface and the chosen field was facilitated by intravenous administration of 0.1 mL 2% fluorescein isothiocyanate (FITC)-labeled dextran (molecular weight 150,000) and by epi-illumination at 460–490 nm using a 520-nm emission filter. Leukocytes were labeled using rhodamine-6G fluorescent dye (0.3 mg/kg) and visualized by epi-illumination at 536–556 nm excitation using a 615-nm emission wavelength. Analysis of leukocyte-endothelium interactions was carried out by examining four randomly selected venular segments (30 to 100 mm in diameter) in each preparation. Rolling leukocytes were defined as the number of cells crossing the venular segment at a speed slower than red blood cells for 1 min. Adherent leukocytes were defined as the total number of leukocytes that were rigidly attached to the endothelium and did not change position during 1 min of observation and expressed as the number of cells/min/100 μm.

### In vitro experimental protocol

Human microvascular endothelial cells (HMEC, ATCC CRL-3243) were routinely cultured in MCDB131 growth medium (Gibco, Waltham, MA), supplemented with 10 ng/mL epidermal growth factor (EGF, Sigma-Aldrich, St. Louis, MO), 1 μg/mL hydrocortisone (Sigma-Aldrich—St. Louis, Missouri, E Sigma-Aldrich, St. Louis, MO, USA), 10 mM L-Glutamine (Gibco—Waltham, MA, USA), fetal bovine serum (FBS) to a final concentration of 10% (Gibco—Waltham, MA, USA), Hepes 20 Mm (Sigma-Aldrich, St. Louis, MO), and penicillin and streptomycin to a final concentration of 2% (Gibco—Waltham, MA, USA). Cells were grown at 37 °C in humidified atmosphere of 5% CO_2_ until reaching 80–90% confluence before passaging. HMECs were used between passages 26 and 31. For experiments, HMECs were incubated for 24 h with complete growth medium and treated with lipopolysaccharide (LPS, 1 μg/mL; Sigma-Aldrich, St. Louis, MO) and heme (40 Mm; Frontier Science, Boston, MA). After 24 h, cells were incubated with MSC-conditioned media (CM), prepared as described elsewhere [9]. Lactate dehydrogenase (LDH) release was measured at 6, 18, and 24 h with the commercially available CytoTox 96 Non-Radioactive Cytotoxicity Assay (Promega, Madison, WI).

### Statistical analysis

We used a one-way analysis of variance with post-hoc Tukey test to assess the differences between more than two groups and Student’s *t* test when data from only two groups were analyzed. Survival curves were derived by the Kaplan-Meier method and compared by the log-rank test. We expressed the data as mean ± SEM and set the significance level to 5%. We performed all tests in GraphPad Prism 6.0 (GraphPad Software, San Diego, CA).

## Results

### Mesenchymal stromal cell phenotypic characterization

In accordance with the International Society for Cellular Therapy [15], we observed that isolated mesenchymal stromal cells (MSCs) were adherent to cell culture flasks, capable of differentiating into adipocytes (Supplementary Fig. 1a), positive for the mesenchymal markers CD90 and CD29, and negative for the hematopoietic markers CD45, CD11b, and CD14 (Supplementary Fig. 1b).

### Mesenchymal stromal cell effects on the experimental cerebral malaria model

**Fig. 1:**
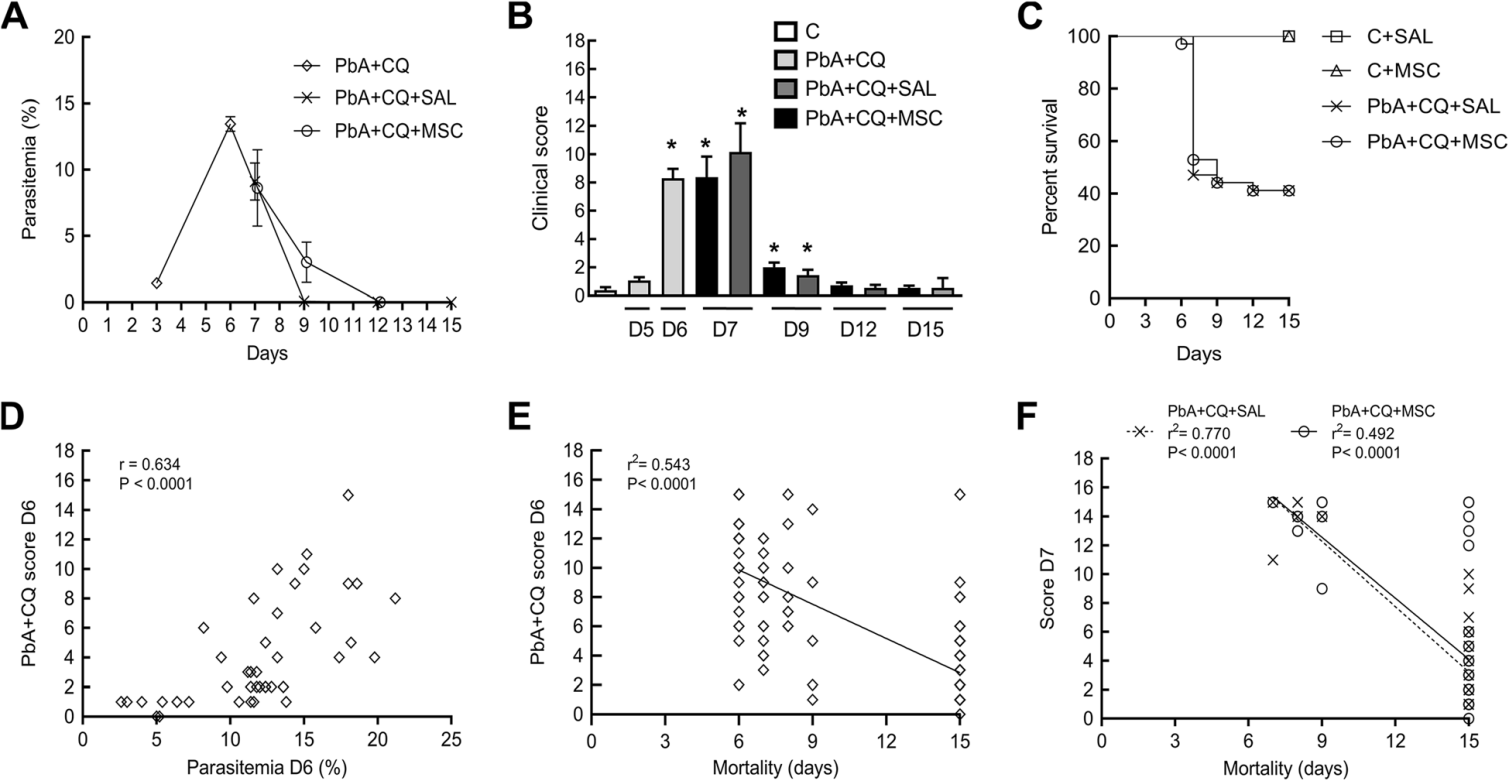
Evaluation of MSC on the experimental cerebral malaria model. **a** Parasitemia in mice at 3, 6, 9, 12, and 15 days post-infection (dpi). **b** Clinical score of mice at 5, 6, 7, 9, 12, and 15 dpi. **c** Kaplan-Meier survival curves of animals. **d** Correlation between parasitemia and clinical score of PbA+CQ mice at 6 dpi. **e** Linear regression analysis between clinical score of PbA+CQ mice at 6 dpi and mortality. **f** Linear regression analysis between clinical score of PbA+CQ+SAL and PbA+CQ+MSC mice at 7 dpi and mortality. C, control; PbA, *Plasmodium berghei* ANKA; CQ, chloroquine; SAL, saline; MSC, mesenchymal stromal cells. Data presented as mean ± SEM (**a**, **b**). *N* = 8–14 mice per group. *P < 0.05 vs. C by one-way analysis of variance

Parasitemia in mice peaked at 6 days post-infection (dpi), coinciding with the start of chloroquine (CQ) treatment. In both PbA+CQ+SAL and PbA+CQ+MSC experimental groups, parasitemia began to decrease from 7 to 15 dpi. MSC therapy neither interfered with the progression of PbA infection nor recovery of animals following chloroquine administration (Fig. 1a). PbA-infected mice presented higher clinical score at 6, 7, and 9 dpi when compared to the non-infected control group (Fig. 1b). MSC-treated mice (PbA+CQ+MSC) had a similar clinical score (Fig. 1b) and similar survival rates (Fig. 1c) when compared to the non-treated group (PbA+CQ+SAL). Correlation analysis was performed between parasitemia and clinical score at 6 dpi in the PbA+CQ group, and a positive association was observed between these two variables (Fig. 1d). Regression analysis was also performed between clinical score at 6 dpi and mortality (Fig. 1e) as well as between the clinical score at 7 dpi and mortality in the PbA+CQ+MSC group (Fig. 1f). Although MSC treatment did not alter survival rates in mice, regression analysis showed that mice from the PbA+CQ+MSC group with a high clinical score at 7 dpi survived until 15 dpi, which was not observed in mice from the PbA+CQ+SAL group (Fig. 1f). The PbA+CQ+SAL and PbA+CQ+MSC groups presented increased spleen weight at 7 and 15 dpi (Supplementary Fig. 2).

### Mesenchymal stromal cells preserved blood-brain barrier integrity and reduced adherent leukocytes

**Fig. 2:**
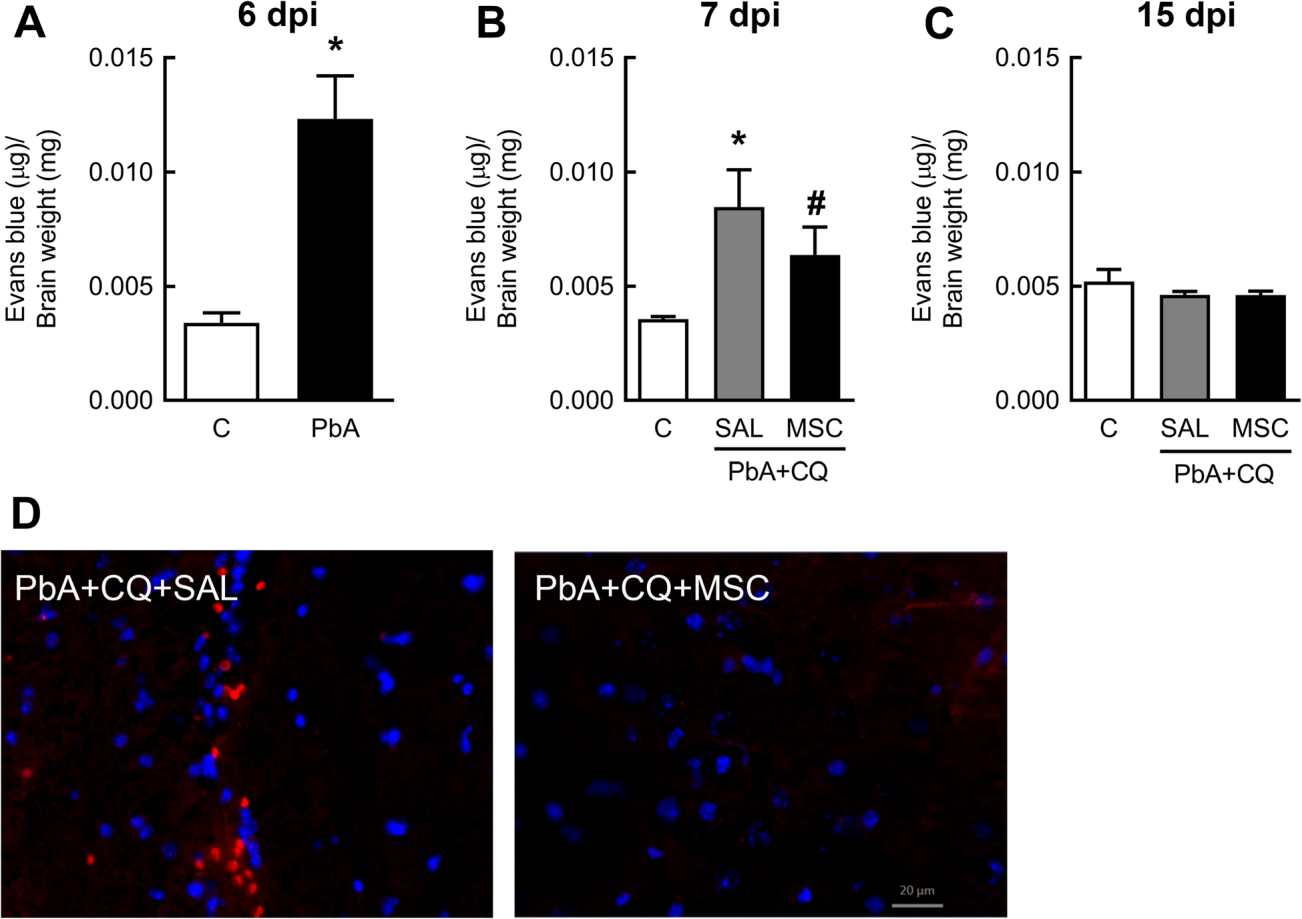
MSCs improved blood-brain barrier integrity. MSC effects on BBB integrity, assessed by Evans blue dye leakage at **a** 6 dpi, **b** 7dpi, and **c** 15 dpi. **d** Representative images demonstrating the presence of erythrocytes in the brain parenchyma of PbA-infected mice with SAL and the absence of them with MSC treatment at 7dpi. DAPI (4′,6-diamidino-2-phenylindole)-stained nucleus in blue and erythrocytes autofluorescence in red. C, control; PbA, *Plasmodium berghei* ANKA; CQ, chloroquine; SAL, saline; MSC, mesenchymal stromal cell. Data presented as mean ± SEM. *N* = 5–8 mice per group. *P < 0.05 vs. C and ^#^*P* < 0.05 vs. PbA+CQ+SAL by *t* test or one-way analysis of variance

**Fig. 3:**
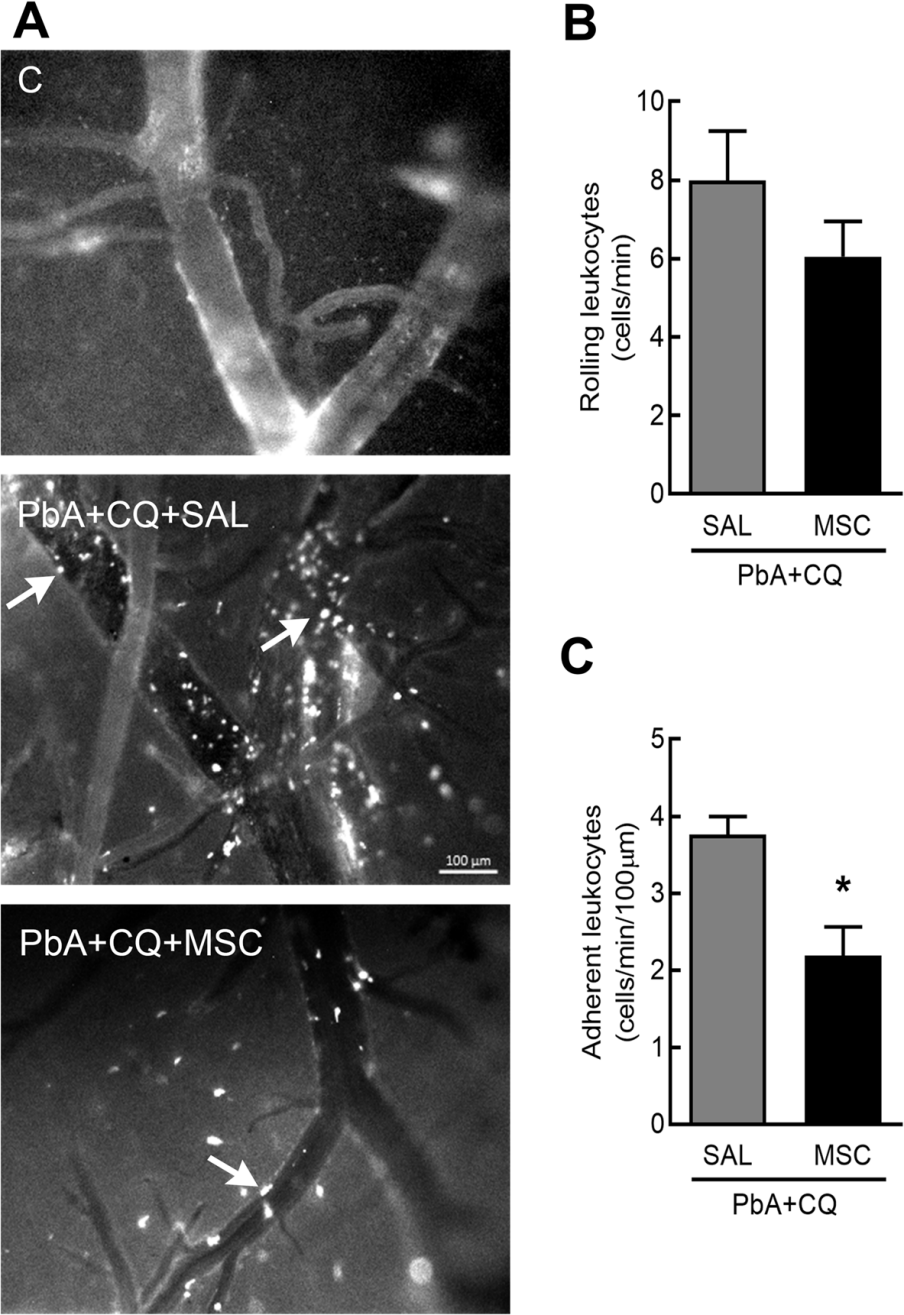
Evaluation of MSCs on rolling and adherent leukocytes in brain microcirculation at 7 dpi. **a** Representative images of leukocyte-endothelium interaction in the brain microcirculation of control and PbA-infected mice with or without treatment with MSC or SAL. **b** Rolling and **c** adherent rhodamine 6G-labeled leukocytes evaluated by intravital fluorescence microscopy on cerebral post-capillary venules. C, control; PbA, *Plasmodium berghei* ANKA; CQ, chloroquine; SAL, saline; MSC, mesenchymal stromal cell. Data presented as mean ± SEM. *N* = 4 mice per group. *P < 0.05 vs. PbA+CQ+SAL by *t* test. *P < 0.05. Scale bar is 100 μm

**Fig. 4:**
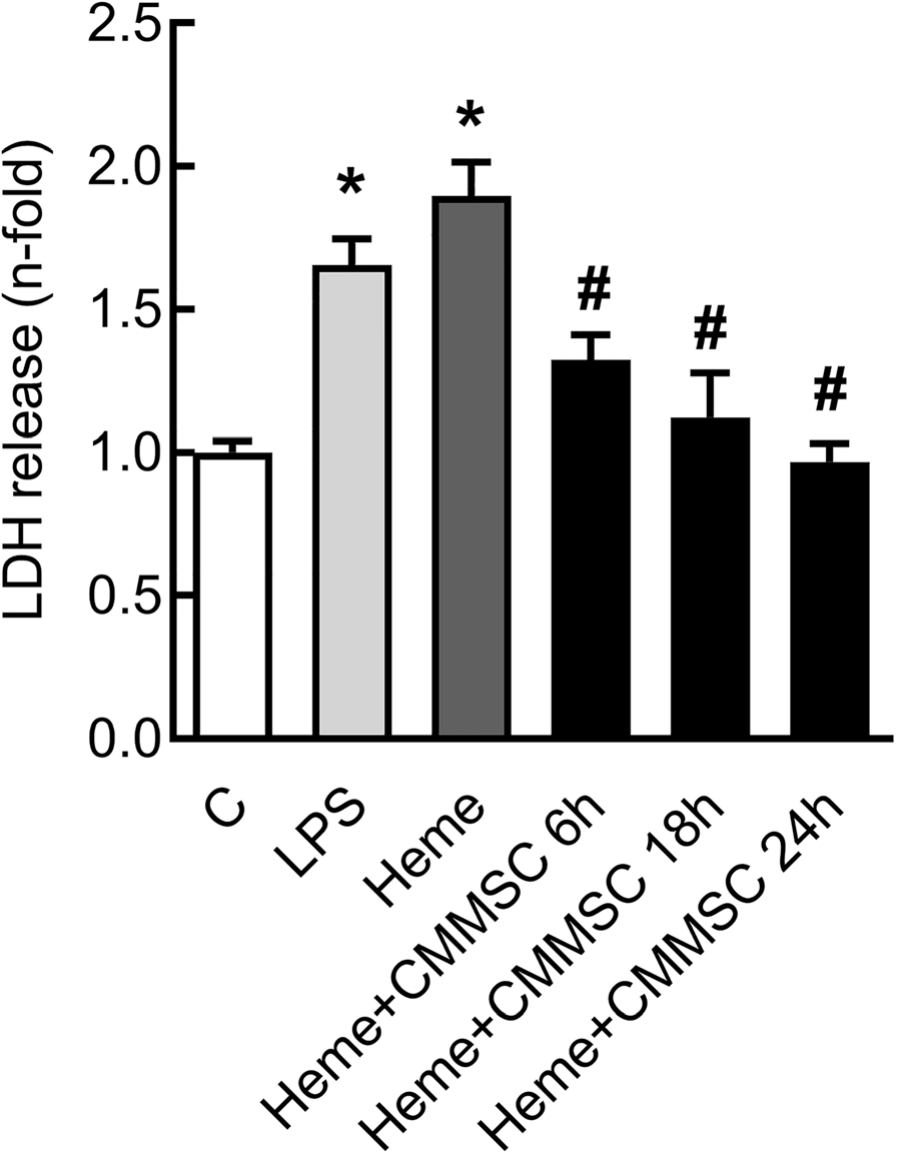
Conditioned media derived from MSCs (CMMSC) reduced lactate dehydrogenase (LDH) release in cultured HMEC cells. HMEC cells were stimulated with lipopolysaccharide (LPS) and heme. CMMSC reduced LDH release at 6, 18, and 24 h. Data presented as mean ± SEM. *n* = 3–12 per group. *P < 0.05 vs. C and ^#^*P* < 0.05 vs. heme by one-way analysis of variance

PbA-infected mice exhibited loss of blood-brain barrier (BBB) integrity compared to the control group at 6 (Fig. 2a) and 7 dpi (Fig. 2b), but not at 15 dpi (Fig. 2c). The PbA+CQ+MSC group showed higher BBB integrity in comparison to the PbA+CQ+SAL group at 7 dpi (Fig. 2b). There was no significant difference between the C+SAL and C+MSC groups (Supplementary Table 1). The presence of erythrocytes in the brain parenchyma was observed only in the PbA+CQ+SAL group (Fig. 2d). To further clarify this point, we evaluated inflammatory cell dynamics and the leukocyte-endothelium interaction in cerebral microcirculation using intravital microscopy by epi-illumination and fluorescence. We observed an expressive number of rolling (Fig. 3a, b) and adhered (Fig. 3a, c) leukocytes in the cerebral post-capillary venules of different calibers, causing stagnation of the blood cells and stasis of the flow in the mice from the PbA+CQ+SAL group (Supplementary video 1) compared to control group (Fig. 3a, Supplementary video 2). MSC treatment alleviated inflammation in cerebral microcirculation, evidenced by a reduction of adhered leukocytes (Fig. 3a, c, Supplementary video 3). Accordingly, conditioned media derived from MSC (CMMSC) reduced lactate dehydrogenase (LDH) release in cultured HMECs stimulated with heme at 6, 18, and 24 h (Fig. 4).

### Mesenchymal stromal cells improved depression-like behavior

**Fig. 5:**
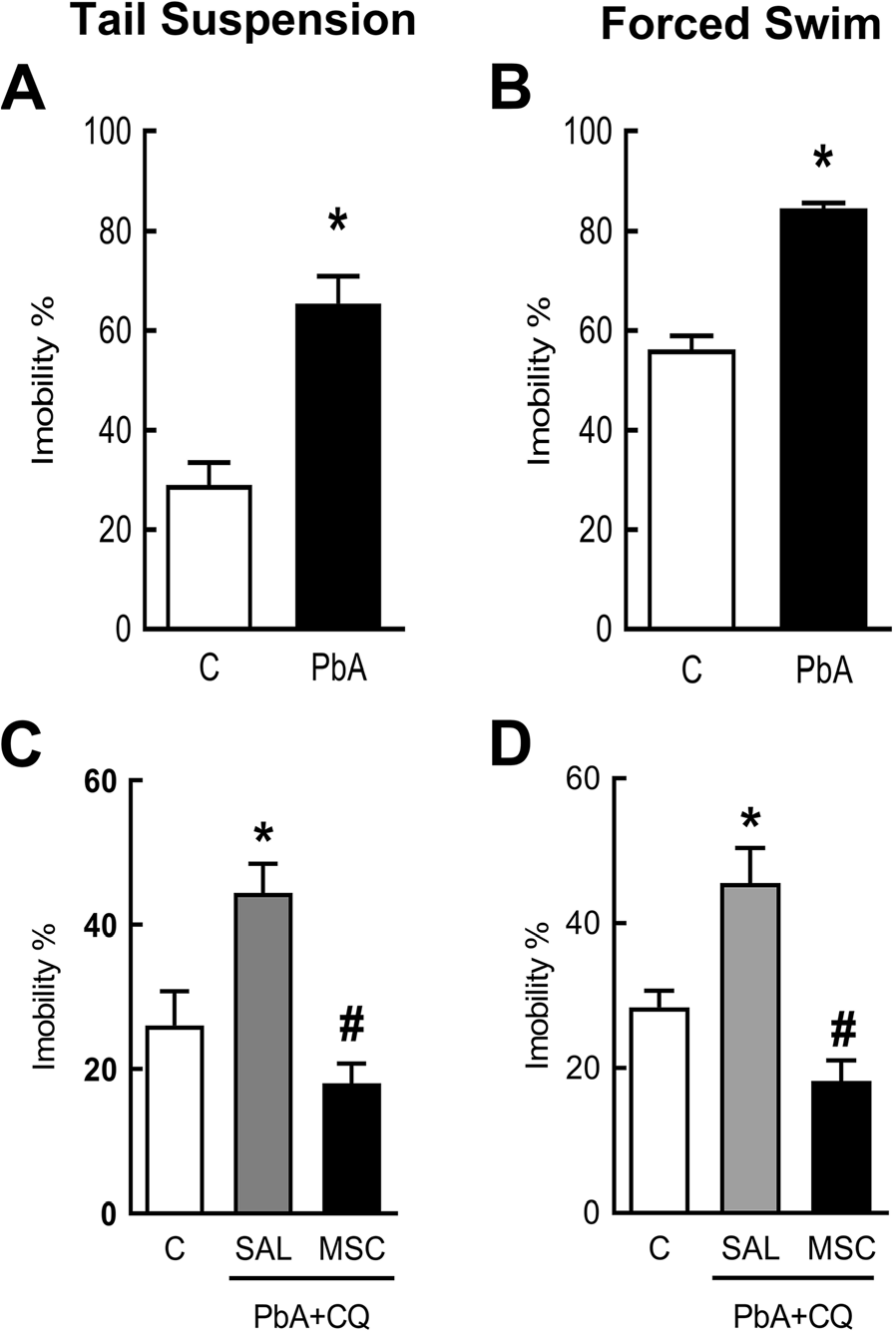
MSCs improved depressive-like behavior in mice 15 dpi. Behavioral tests were performed 15 dpi in surviving mice. Percentage of total immobility time in the tail suspension test at **a** 5 dpi and **c** 15 dpi. Percentage of total immobility time in the forced swim test at **b** 5 dpi and **d** 15 dpi. C, control; PbA, *Plasmodium berghei* ANKA; CQ, chloroquine; SAL, saline; MSC, mesenchymal stromal cell. Data presented as mean ± SEM. *N* = 5 mice per group. *P < 0.05 vs. C and ^#^*P* < 0.05 vs. PbA+CQ+SAL by *t* test or one-way analysis of variance

PbA-infected mice showed more immobility in the tail suspension (Fig. 5a) and forced swimming (Fig. 5b) tests at 5 dpi compared to control mice. At 15 dpi, MSC therapy reversed depression-like behavior in both tests (Fig. 5c, d) compared to mice from the PbA+CQ+SAL group. There was no significant difference between C+SAL and C+MSC groups (Supplementary Table 1).

### Mesenchymal stromal cell effects on BDNF and Neuroinflammation

**Fig. 6:**
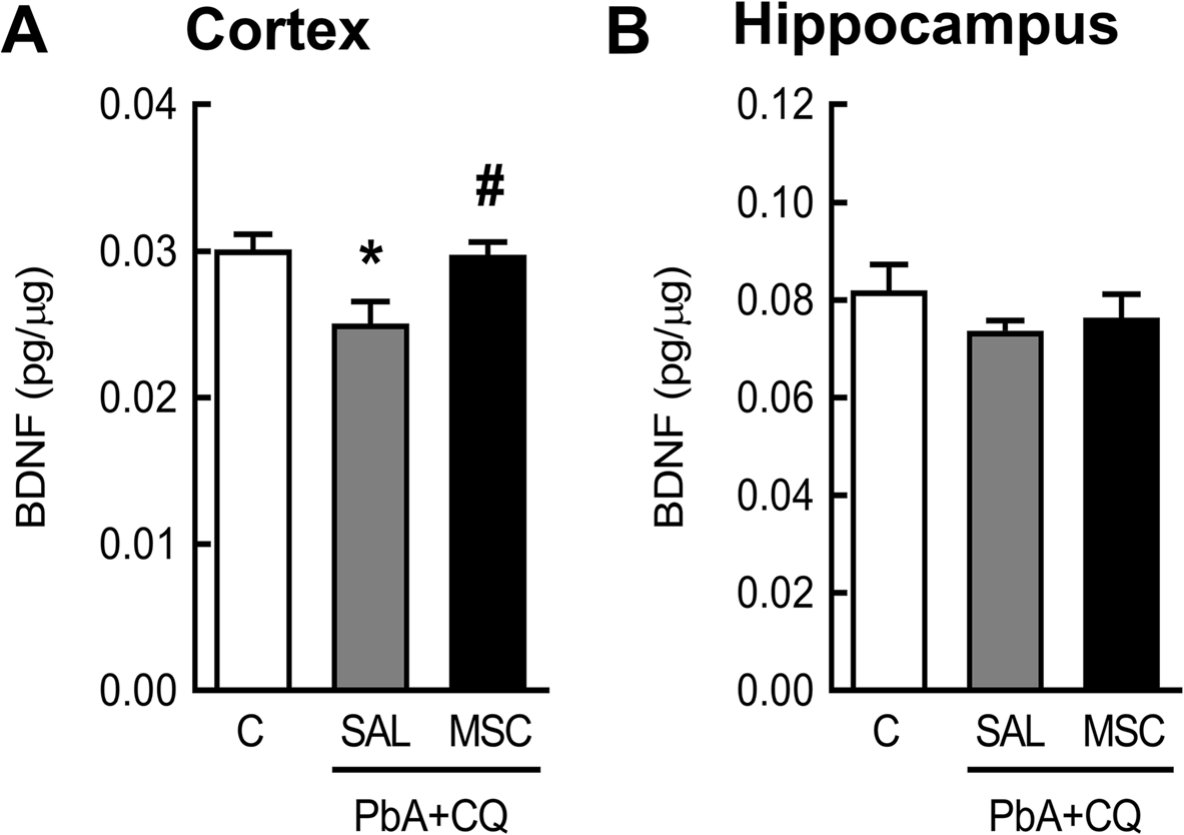
Effects of MSCs on BDNF at 7 dpi. BDNF protein levels in the **a** cortex and **b** hippocampus. C, control; PbA, *Plasmodium berghei* ANKA; CQ, chloroquine; SAL, saline; MSC, mesenchymal stromal cell. Data presented as mean ± SEM. *N* = 4–11 mice per group, *P < 0.05 vs. C and ^#^*P* < 0.05 vs. PbA+CQ+SAL by one-way analysis of variance

**Fig. 7:**
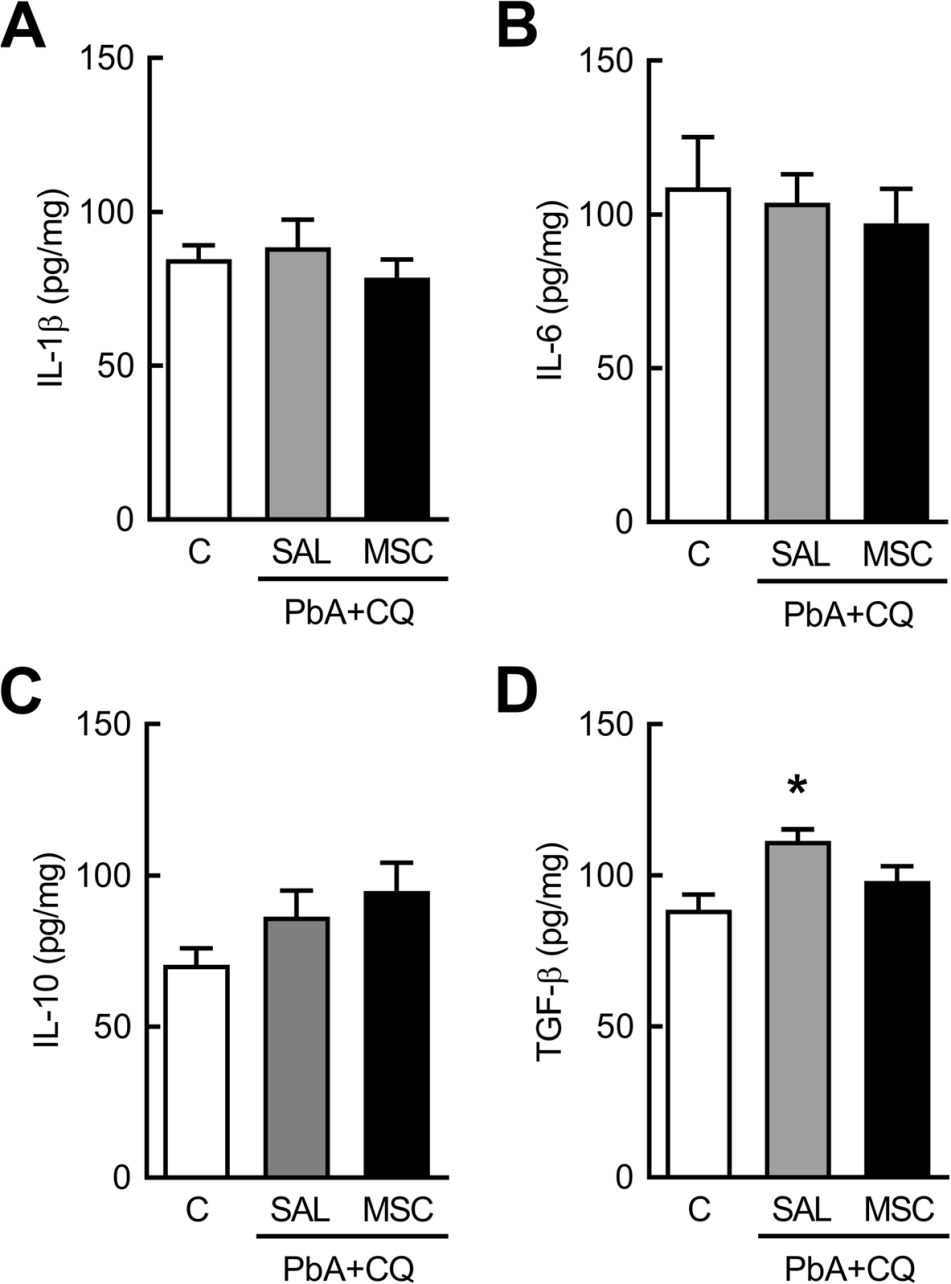
Effects of MSCs on neuroinflammation 15 dpi. **a** interleukin (IL)-1β, **b** IL-6, **c** IL-10, and **d** transforming growth factor (TGF)-β brain protein levels. C, control; PbA, *Plasmodium berghei* ANKA; CQ, chloroquine; SAL, saline; MSC, mesenchymal stromal cell. Data presented as mean ± SEM. *N* = 5–9 mice per group, *P < 0.05 vs. C by one-way analysis of variance

MSC therapy restored BDNF levels in the cortex at 7 dpi compared to mice from the PbA+CQ+SAL group (Fig. 6a). There was no significant difference between C+SAL and C+MSC groups (Supplementary Table 1). At 15 dpi, mice from the PbA+CQ+SAL group showed no differences in IL-1β, IL-6, and IL-10 secretion levels (Fig. 7a–c) when compared to control mice, while TGF-β levels were increased in this group (Fig. 7d).

## Discussion

In this study, we show that mesenchymal stromal cells, as an adjuvant therapy for brain damage, reduced leukocyte-endothelium interactions in the brain microvasculature, mitigated BBB damage, and restored BDNF levels in the cortex of PbA-infected C57BL/6 mice. Furthermore, MSC administration improved depressive-like behavior 15 days after infection in mice that survived cerebral malaria. To the best of our knowledge, this was the first study to demonstrate the positive effects of MSCs as an adjuvant therapy for brain damage in an experimental model of malaria.

We chose to initiate chloroquine administration 6 days after PbA infection as the antimalarial therapy. The strong correlation between parasitemia and clinical score 6 days post-infection enabled the evaluation of the severity and progression of the disease. This analysis allowed us to use the clinical score as a cutoff point for infection, making the study more reliable and less susceptible to outliers. We excluded data from mice that presented a clinical score below 4, considered as abnormal progression of the infection. Also, using clinical score and survival linear regression analysis, we identified that mice with higher scores are more likely to die before day 15.

The MSC treatment did not modulate parasitemia, clinical score, or survival rates in the infected mice. Although MSC therapy did not improved survival rates, we noticed that mice in the PbA+CQ+MSC group with high scores at day 7 post-infection managed to reach day 15, which did not occur in the PbA+CQ+SAL group. Our results differ from Souza and colleagues, who show that MSCs decreased parasitemia and improved survival, which could be explained by their use of a different protocol. They administered MSCs only 24 h after PbA infection and performed analysis 4 days after PbA infection [16], while, in the present study, we administered MSCs on the sixth day post-infection, when mice presented both high parasitemia and clinical score, and analysis was performed at 7 and 15 days after infection. Similarly, in a previous study from our group, MSCs administered 6 h after sepsis induction did not improve clinical score and survival rates [9]. However, when MSCs was administered before sepsis induction, we observed improvement in these parameters (unpublished data), reinforcing that timing is relevant for MSC therapy.

The pathogenesis of cerebral malaria comprises cytoadherence and sequestration of infected red blood cells in the cerebral microvasculature and an inflammatory response to the parasite in the central nervous system. The increase of inflammatory signals elicits vascular leakage and might contribute to microcirculatory dysfunction and neurological symptoms [17]. The activation of multiple cell death pathways in endothelial cells directly mediated by activated T lymphocytes disrupts blood-brain barrier integrity [18]. The T lymphocyte response persists after the resolution of the infection following antimalarial treatment and might be involved in later outcomes in experimental cerebral malaria [19]. We observed BBB disruption at days 6 and 7 post-infection and a higher level of LDH release when HMEC cells were stimulated with heme, indicating increased cell death due to heme from the presence of PbA-infected red cells in the brain. Heme is produced by malaria trophozoites during intra-erythrocytic development and has an important role in the pathophysiology of cerebral malaria [20, 21]. Increased levels of free heme contribute to inflammation, tissue damage, and BBB dysfunction [22, 23] and lead to apoptosis of brain endothelial cells in vitro [20]. Increased number of leukocytes and platelets at the site of parasite sequestration in the brain and upregulation of chemokines and cytokines are present in malaria patients [24, 25] and in murine models of cerebral malaria [26, 27]. Accordingly, we observed an increase in adherent leukocytes in the brain microvasculature in PbA+CQ+SAL mice. Mesenchymal stromal cell therapy has beneficial effects in infectious diseases. In a previous study, mesenchymal stromal cell therapy had beneficial effects in infectious diseases. We observed that MSC administration prevented BBB damage and cognitive and behavioral alterations, as well as reduced astrogliosis in an experimental model of CLP-induced sepsis [9]. Herein, we were the first to demonstrate that intravenous infusion of a single dose of MSC mitigated BBB dysfunction and reduced leukocyte adhesion to brain microvasculature in PbA-infected mice. In addition, conditioned media derived from MSCs increased cell viability in HMEC cultured cells stimulated with heme. According to the literature, MSCs are primarily entrapped in the lungs after intravenous delivery; however, the amount of bone marrow cells trapped within the pulmonary and brain vasculature after systemic administration is small [28–32], suggesting that cellular migration and engraftment are not required for positive effects to occur; instead, MSCs appear to act predominantly through a paracrine mechanism [33].

In this work, treatment with MSCs significantly reduced leukocyte adhesion in post-capillary venules, even though the decrease in rolling was not shown to be statistically significant. One explanation may be the possible action of the MSC on adhesion molecules, such as intercellular adhesion molecule (ICAM)-1, but not on selectins. The former are key elements for rigid leukocyte adhesion to the vascular endothelium. On the other hand, selectins promote weak binding of leukocytes to the endothelium, a feature of the rolling process. Corroborating our finding, a recent study demonstrated that human placenta MSCs can exert an anti-inflammatory effect. Authors showed significantly attenuated expression of NF-κB messenger RNA, inhibiting overactivation of NF-κB and further reducing the generation of tumor necrosis factor (TNF)-α and ICAM-1 by MSCs in an inflammatory mouse model of kidney injury [34].

Behavioral disorders are likely to become neglected in patients with more severe neurological damage, such as cognitive impairments and motor function deficits, frequently observed in cerebral malaria survivors [2, 35]. Individuals infected with *Plasmodium falciparum* present a significant increase in anxiety and depression [4, 36]. Several studies in experimental models of malaria also demonstrated anxiety and depression-like behavior alterations [37–39]. We also observed an increase in depression like-behavior in PbA-infected mice, while MSC treatment mitigated this behavioral alteration. We performed tail suspension and forced swim tests on days 5 and 15 after PbA infection. Both tests are commonly used in the study of potential antidepressant drugs. They assume that if an animal cannot escape or hold onto nearby surfaces, it will first make efforts to escape but ultimately will exhibit immobility that may be considered a measure of behavioral despair [12, 13]. The underlying MSC mechanism of action may involve BDNF protein levels in the cortex. Some cortical brain areas are implicated in depression [40] and decreased expression of neurotrophins and impaired neurogenesis were detected in animal models of depression [41, 42]. BDNF modulates survival of neurons and plasticity mechanisms in learning and memory [43] and plays an important role in the pathogenesis of depression [41, 44, 45]. The activation of the immune system during disease or stress conditions may inhibit neurotrophin release and contribute to the deleterious effects in adult neurogenesis and cognitive function [46, 47]. Reduced BDNF expression levels are associated with cognitive and behavioral disorders in neurological disorders, such as cerebral malaria [48–50]. Previous studies have showed reduced BDNF mRNA expression in the frontal cortex and the hippocampus [37] and in various brain regions, including the thalamus, hypothalamus, cerebellum, and brainstem in PbA-infected mice [51]. The downregulation of BDNF may be a point of no return for the neurological damages observed in cerebral malaria. In the present study, MSC treatment restored BDNF levels, in accordance with others that demonstrated that MSC therapy increases BDNF levels in the brain and elicits functional improvement [52, 53]. Although MSCs secretes BDNF, this might not be the only mechanism of action. MSC protective effects on the BBB may lead to a reduction in the activation of microglia and astrocytes that can modulate neuroinflammation and the development of the neurological damage [8, 9]. BBB dysfunction has been correlated to the influx of proinflammatory mediators into the brain tissue and consequent depression-like behavior [54–57].

Since the malaria parasite is not neurotropic, the neurological sequelae may be due to a neuroinflammatory effects generated in response to infection [17, 19]. The imbalance of pro- and anti-inflammatory cytokines in the brain is related to the pathogenesis of cerebral malaria. Most studies evaluate cytokines and chemokines in the acute phase of the disease [5, 58], whereas, we chose to analyze inflammatory mediators at day 15, when depression-like behavior was still observed in mice. We then observed that IL-1β, IL-6, and IL-10 protein expression levels were already restored in PbA-infected mice brains and that TGF-β protein expression levels were increased in the PbA+CQ+SAL group. TGF-β modulates the inflammatory response in the central nervous system as well as the proliferation of microglia and astrocytes [59, 60]. In addition, TGF-β upregulation may be detrimental to regeneration. Members of the TGF-β family inhibit neurogenesis by blocking proliferation of precursor cells in the adult brain [61], and TGF-β intracerebroventricular injection reduced the number of proliferating cells in the hippocampus and subventricular zone in adult mice [62].

## Conclusion

In mice that survived experimental cerebral malaria, a single dose of MSCs conferred a protective effect on the blood-brain barrier, reduced the number of adherent leukocytes, and restored BDNF protein levels within 24 h of administration. In addition, MSC therapy improved depressive-like behavior 15 days after PbA infection. Thus, we believe that MSC administration as an adjuvant therapy for infectious diseases, such as malaria, may be a promising treatment, especially for neurological damages.

## Supplementary information

**Additional file 1: Supplementary Fig. 1.** MSC phenotypic characterization. **a** Adipogenic (Oil red O staining) differentiation of mesenchymal stromal cells (MSCs) isolated from 8-week-old C57BL/6 mice femur and tibia bone marrow. **b** Representative FSC vs. SSC dot plot showing the gate for MSCs derived from C57BL/6 mice, between the third and fifth passages. Histograms in red or green showing expression of mesenchymal markers CD29 and CD90; isotype control or only anti-rat Alexa Fluor 488 antibody in gray. MSCs were negative for expression of the hematopoietic markers CD45, CD11b, and CD14.

**Additional file 2: Supplementary Fig. 2.** Evaluation of spleen weight in mice. Analysis of spleen and body weight at 7 and 15 dpi. Abbreviations: C, control; PbA, *Plasmodium berghei* ANKA; CQ, chloroquine; SAL, saline; MSC, mesenchymal stromal cells. Data presented as mean ± SEM. *N* = 8–15 mice per group, *P < 0.05 vs. C by one-way analysis of variance.

**Additional file 3: Supplementary Table 1.** C+SAL vs. C+MSC groups.

**Additional file 4: Supplementary video 1**.

**Additional file 5: Supplementary video 2**.

**Additional file 6: Supplementary video 3**.

## Data Availability

All data generated or analyzed during this study are included in this published article and its supplementary information files.

## Abbreviations

BBB: Blood-brain barrier
BDNF: Brain-derived neurotrophic factor
C: Control
CMMSC: Conditioned media derived from mesenchymal stromal cells
CQ: Chloroquine
dpi: Days post-infection
IL: Interleukin
LDH: Lactate dehydrogenase
LPS: Lipopolysaccharide
MSC: Mesenchymal stromal cells
PbA: *Plasmodium berghei* ANKA
SAL: Saline
