# Supplementary figures and images for: Mesenchymal stromal cells protect against vascular damage and depression-like behavior in mice surviving cerebral malaria
Source: Stem Cell Res Ther. 2020 Aug 26;11:367. doi: 10.1186/s13287-020-01874-6 (PMC7448996; doi:10.1186/s13287-020-01874-6)

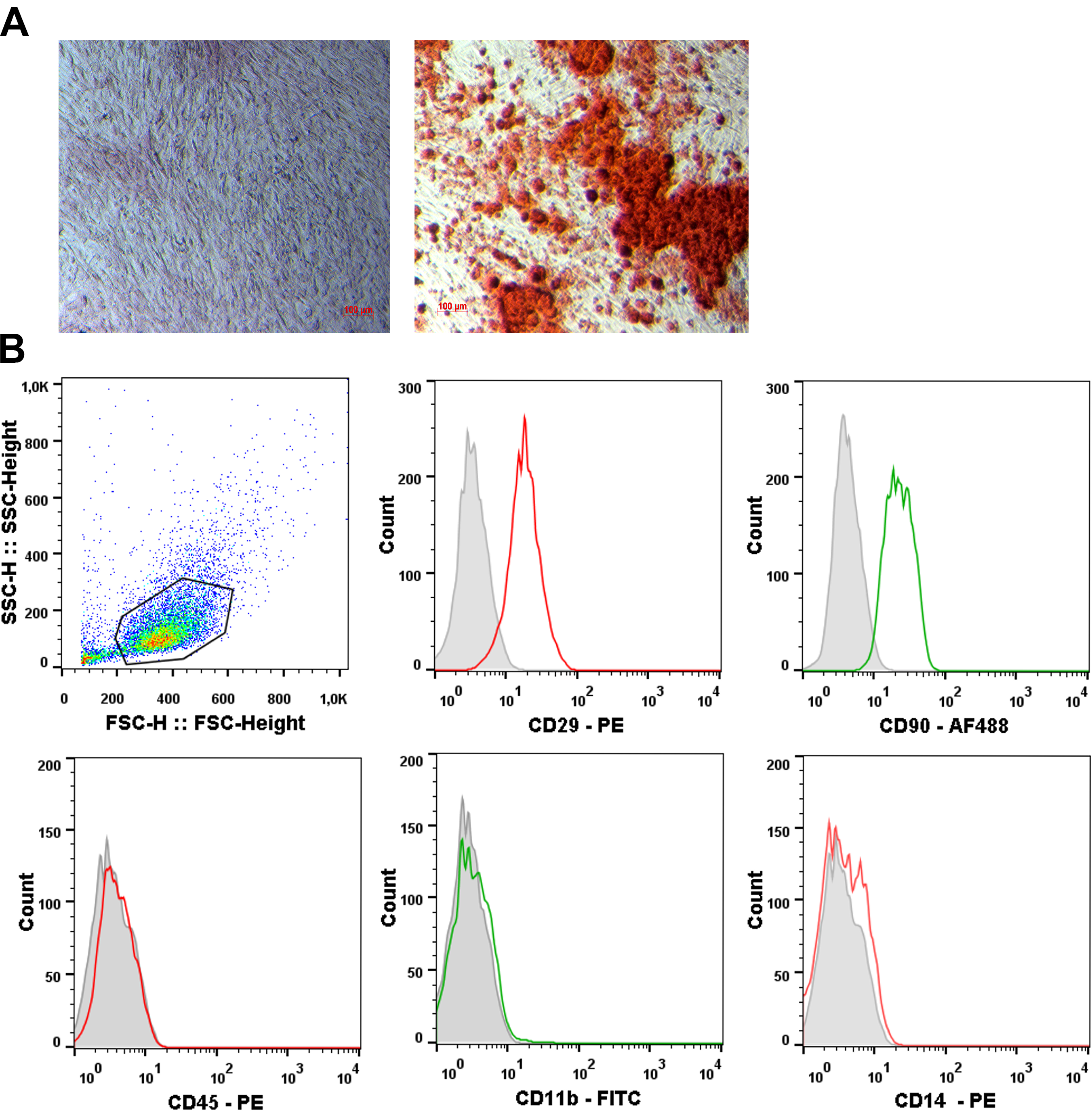

Supplement: Supplementary file 1 — Additional file 1: Supplementary Fig. 1. MSC phenotypic characterization. a Adipogenic (Oil red O staining) differentiation of mesenchymal stromal cells (MSCs) isolated from 8-week-old C57BL/6 mice femur and tibia bone marrow. b Representative FSC vs. SSC dot plot showing the gate for MSCs derived from C57BL/6 mice, between the third and fifth passages. Histograms in red or green showing expression of mesenchymal markers CD29 and CD90; isotype control or only anti-rat Alexa Fluor 488 antibody in gray. MSCs were negative for expression of the hematopoietic markers CD45, CD11b, and CD14. [file 13287_2020_1874_MOESM1_ESM.tif]

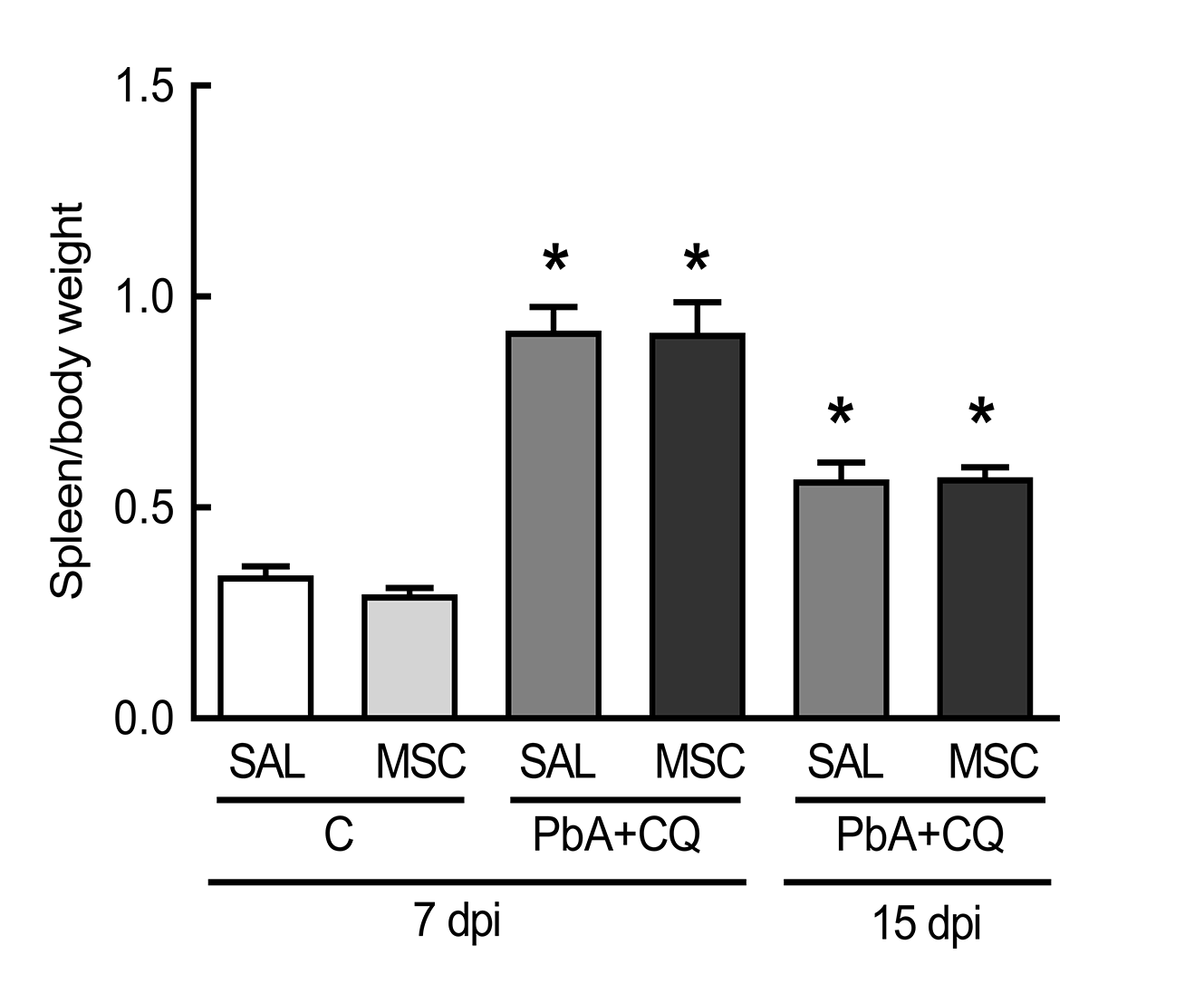

Supplement: Supplementary file 2 — Additional file 2: Supplementary Fig. 2. Evaluation of spleen weight in mice. Analysis of spleen and body weight at 7 and 15 dpi. Abbreviations: C, control; PbA, Plasmodium berghei ANKA; CQ, chloroquine; SAL, saline; MSC, mesenchymal stromal cells. Data presented as mean ± SEM. N = 8–15 mice per group, *P < 0.05 vs. C by one-way analysis of variance. [file 13287_2020_1874_MOESM2_ESM.tif]
