# Supplementary material for: Mesenchymal stromal cells protect against vascular damage and depression-like behavior in mice surviving cerebral malaria
Source: Stem Cell Res Ther. 2020 Aug 26;11:367. doi: 10.1186/s13287-020-01874-6 (PMC7448996; doi:10.1186/s13287-020-01874-6)
Supplement: Supplementary file 3 — Additional file 3: Supplementary Table 1. C+SAL vs. C+MSC groups. [file 13287_2020_1874_MOESM3_ESM.docx]

**Supplementary Table 1:** C+SAL vs. C+MSC groups.

|  | **C+SAL** | **C+MSC** | ***p*** |
| --- | --- | --- | --- |
| Evans blue/Brain weight at 7 dpi (μg/mg) | 0.0043 ± 0.0006 | 0.0037 ± 0.0002 | 0.3226 |
| Evans blue/Brain weight at 15 dpi (μg/mg) | 0.0052 ± 0.0005 | 0.0056 ± 0.0005 | 0.6212 |
|  |  |  |  |
| Cortex BDNF (pg/μg) | 0.0287 ± 0.0017 | 0.0314 ± 0.0020 | 0.3273 |
| Hippocampus BDNF (pg/μg) | 0.0819 ± 0.0053 | 0.0768 ± 0.0020 | 0.4199 |
|  |  |  |  |
| Immobility Forced Swim (%) | 33.83 ± 5.72 | 37.68 ± 12.47 | 0.3402 |
| Immobility Tail Suspension (%) | 26.06 ± 4.74 | 35.13 ± 6.93 | 0.8200 |

*T-*test. Data given as mean ± SEM. Evans blue leakage was measured at 7 and 15 days, BDNF levels at 7 days, and behavioral tests at 15 days after PbA infection in surviving animals.
